# Peutz–Jeghers syndrome with concurrent lobular endocervical glandular hyperplasia and sex cord tumor with annular tubules: a case report

**DOI:** 10.3389/fmed.2026.1785608

**Published:** 2026-05-01

**Authors:** Min Yin, Chunli Lu, Lei Cheng

**Affiliations:** 1 Department of Gynecology, Qilu Hospital of Shandong University, (Qingdao), Cheeloo College of Medicine, Shandong University, Qingdao, China; 2 Department of Spine Surgery, Qilu Hospital of Shandong University (Qingdao), Cheeloo College of Medicine, Shandong University, Qingdao, China

**Keywords:** gynecology, lobular endocervical glandular hyperplasia, Peutz–Jeghers syndrome, sex-cord tumor with annular tubules, STK11

## Abstract

**Background:**

Peutz–Jeghers syndrome (PJS) is an autosomal dominant disorder characterized by mucocutaneous pigmentation, gastrointestinal hamartomatous polyps, and an increased risk of specific neoplasms, including distinctive gynecological tumors.

**Case presentation:**

This study reports a rare case of a 25-year-old woman with a confirmed diagnosis of PJS who presented with watery vaginal discharge. Gynecological evaluation revealed concurrent cervical and ovarian masses. Histopathology of the cervical lesion demonstrated lobular endocervical glandular hyperplasia (LEGH), while the ovarian mass was identified as a sex-cord tumor with annular tubules (SCTATs), exhibiting both simple and complex ring-like tubules surrounding hyaline cores.

**Conclusion:**

This case represents an uncommon co-occurrence of LEGH and SCTATs in a single PJS patient, highlighting the broad spectrum of PJS-associated gynecologic pathology. This study underscores the need for early and regular gynecological surveillance in women with PJS.

## Introduction

Peutz–Jeghers syndrome (PJS) is a rare autosomal-dominant hereditary polyposis syndrome characterized by gastrointestinal hamartomas and mucocutaneous pigmentations, which is caused by a germline mutation in the serine/threonine kinase 11 (*STK11*) gene. *STK11* encodes a tumor-suppressor kinase that plays a crucial role in cell cycle regulation (1). Individuals with PJS have an elevated lifetime risk of developing various malignancies. Gynecological manifestations of PJS include lobular endocervical glandular hyperplasia (LEGH), gastric-type endocervical adenocarcinoma (G-EAC), and sex-cord tumor with annular tubules (SCTATs) (2). LEGH was initially considered benign and histologically similar to minimal deviation adenocarcinoma (MDA), but later recognized as a potential precursor lesion of malignancy (3). SCTATs are a rare ovarian sex-cord–stromal tumor that occurs in two subtypes, namely PJS-associated and sporadic SCTATs. PJS-related SCTATs account for 36% of overall SCTAT cases and are typically benign, whereas 20% of sporadic SCTATs may exhibit malignant potential (3). This study reports a rare case of a 25-year-old woman with PJS presenting with concurrent LEGH and SCTATs.

## Case report

**Figure 1:**
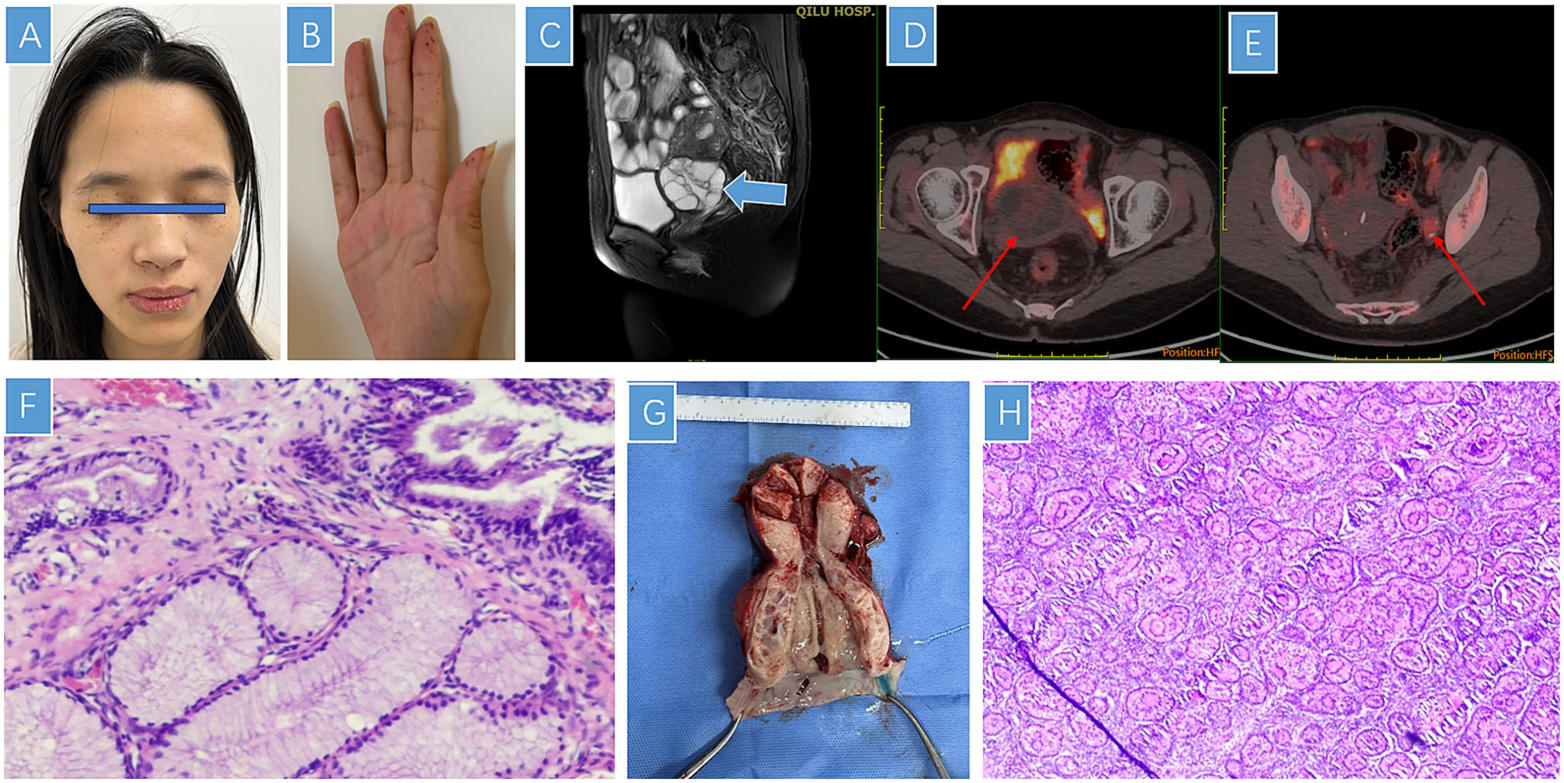
Mucocutaneous pigmentations in the patient on the face **(A)** and fingers **(B)**. MRI findings show a multilocular cystic mass with high signal intensity in the uterine cervix **(C)**. PET/CT demonstrates low-density cystic lesions in the uterine cervix with slightly increased uptake (maximum standardized uptake value [SUVmax]: 1.97) **(D)** and a calcified mass in the left adnexa (SUVmax: 2.70–5.09) **(E)**. Microscopic findings of lobular endocervical glandular hyperplasia show cystic glands surrounded by multiple lobules of benign-appearing endocervical glands, without stromal reaction **(F)**. The inside view of the enlarged uterine cervix shows multiple cystic cavities with smooth walls, containing a large amount of clear watery mucous fluid **(G)**. The ovarian tumor exhibited annular tubules separated by stroma, with an eosinophilic core within the tubules **(H)**.

**Figure 2:**
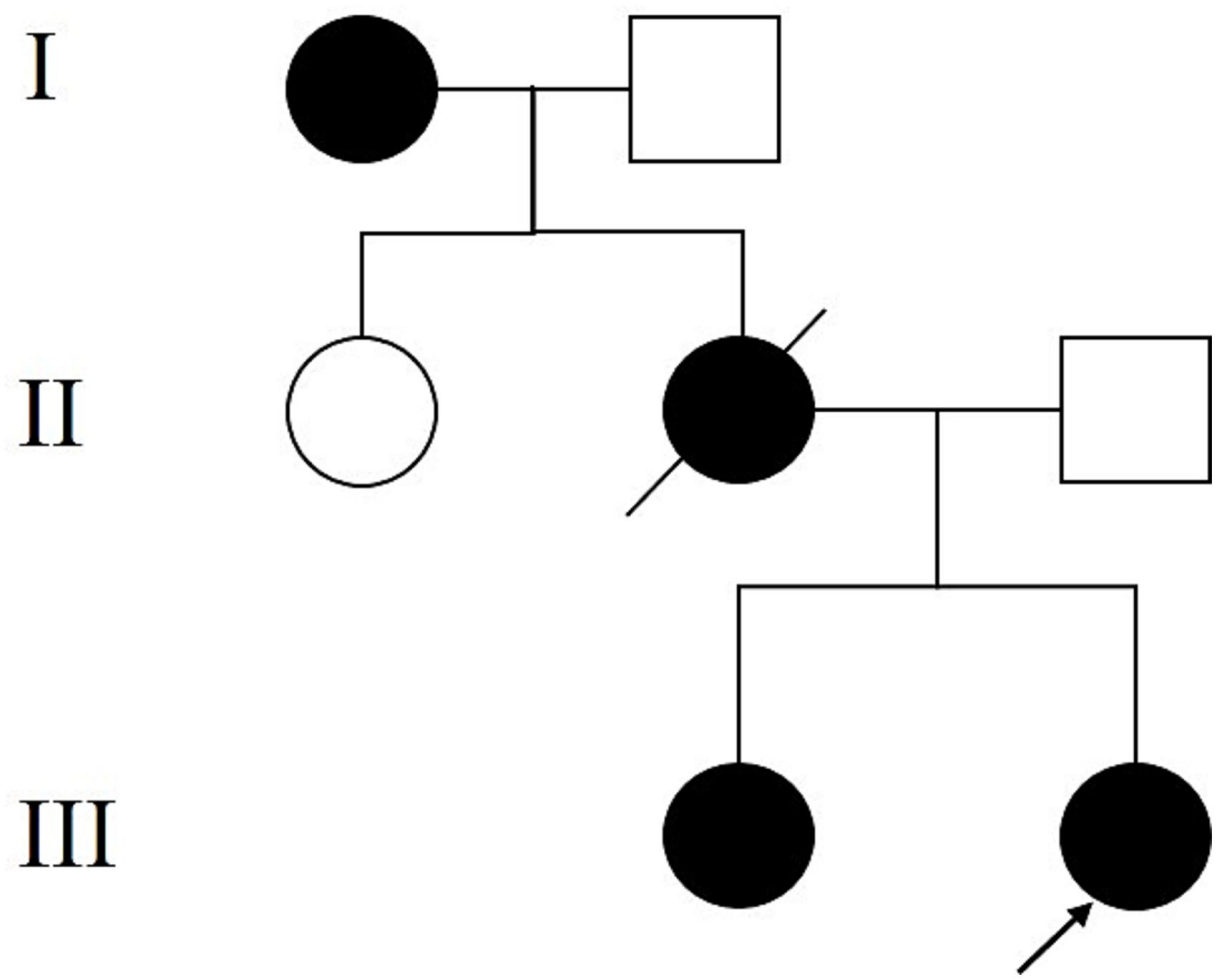
Three-generation pedigree of the patient with PJS. Filled symbols indicate affected individuals; arrows indicate the proband.

A 25-year-old nulligravid woman without a history of sexual intercourse presented with watery vaginal discharge. Physical examination revealed mucocutaneous pigmentations on the face (Figure 1A), buccal mucosa, and fingers (Figure 1B). She had a history of intestinal intussusception at 13 years of age, which was treated with partial colectomy. Pathological examination revealed multiple adenomatous polyps in the small intestine. She subsequently underwent multiple endoscopic polypectomies. Histopathological examination of the small intestinal polyps revealed typical hamartomatous polyps characterized by branching smooth muscle bundles and disorganized mucosal glands, consistent with Peutz–Jeghers-type hamartomas. At 22 years of age, she underwent dilatation and curettage (D&C) for abnormal uterine bleeding, and the specimen revealed endometrial atypical hyperplasia. She was treated with oral megestrol for 1 year, followed by the insertion of a levonorgestrel-releasing intrauterine device (LNG-IUD). At 24 years of age, a duodenal tumor was detected and resected through laparoscopic pancreatoduodenectomy, and the duodenal adenocarcinoma was a well-differentiated adenocarcinoma with an invasive growth pattern, without signet-ring cell components or high-grade microsatellite instability, in line with the gastrointestinal malignancies commonly observed in PJS patients. The family history was notable for similar mucocutaneous pigmentations in her mother and sister. Her mother succumbed to advanced ovarian cancer, and her sister was diagnosed with intestinal polyps and endometrial atypical hyperplasia, while her father was clinically healthy. A three-generation pedigree is presented in Figure 2, showing affected family members with mucocutaneous pigmentation, intestinal polyps, ovarian cancer, and endometrial lesions. Genetic testing was performed using the next-generation sequencing (NGS)-based targeted gene panel on peripheral blood leukocyte DNA. A heterozygous germline mutation with loss of heterozygosity (LOH) in STK11 exons 1–9 was identified, confirming a germline event consistent with the diagnosis of PJS. Pelvic examination revealed a cervical mass confined to the uterine cervix without invasion of adjacent tissues. Doppler ultrasonography showed a 5.2 × 4.2 cm multilocular cystic mass in the uterine cervix and a 3.7 × 2.0 cm heterogeneous echoic mass in the left adnexa. Magnetic resonance imaging (MRI) revealed an enlarged uterine cervix with multilocular cystic lesions (Figure 1C). Positron emission tomography/computed tomography (PET/CT) demonstrated low-density cystic lesions in the uterine cervix with slightly increased uptake (maximum standardized uptake value) (SUVmax: 1.97; Figure 1D) and a calcified mass in the left adnexa (SUVmax: 2.70–5.09; Figure 1E). Serum levels of carbohydrate antigen 125 (CA125) and carbohydrate antigen 199 (CA199) were within the normal reference ranges.

Furthermore, cervical biopsy and hysteroscopic examination were performed. Endometrial biopsy revealed decidual-like stromal changes with no evidence of atypical hyperplasia, and cervical histopathological examination revealed lobular mucinous gland hyperplasia morphologically resembling pyloric glands. Glandular nuclei were enlarged, elongated, and hyperchromatic, with basal localization and no epithelial tufting (Figure 1F). Immunohistochemical staining showed positivity for MUC-6 (+), p16 (focal +), carcinoembryonic antigen (CEA) (−), Pax-8 (focal +), CD10 (focal interstitial +), and Ki-67 in 1–2% of cells. These findings were consistent with the diagnosis of LEGH.

As MDA develops in 15–30% of patients with PJS, and LEGH is considered a potential precursor lesion of MDA (3), the possibility of MDA could not be excluded. After comprehensive counseling, the patient opted for a hysterectomy due to her lack of desire to preserve fertility. She underwent transabdominal hysterectomy, bilateral salpingectomy, and left ovarian tumor resection. Gross pathological examinations revealed multiple mucus-filled cysts in the uterine cervix (Figure 1G). Final pathological examination confirmed LEGH with negative surgical margins. A small amount of fragmented endometrial tissue was observed within the intrauterine blood clot. The glandular tissue was sparse, and the stromal tissue exhibited decidual-like changes. Focal infiltration of acute and chronic inflammatory cells was present. No atypical hyperplasia of endometrial tissue was detected in the specimen examined. The left ovarian tumor exhibited characteristic SCTAT morphology, with annular tubules surrounding hyaline cores and calcification (Figure 1H). The immunohistochemical staining showed positivity for calretinin, inhibin-A, steroidogenic factor-1 (SF-1), CD56 (focal), Wilms tumor 1 (WT-1), and pan-cytokeratin (CKpan) (focal), and negativity for epithelial membrane antigen (EMA), CK5/6, estrogen receptor (ER), and progesterone receptor (PR). The Ki-67 proliferation index was 5%, thereby confirming the diagnosis of SCTATs. The right ovary was examined intraoperatively, and it appeared entirely normal on gross inspection. The patient had an uneventful recovery and was discharged 10 days after surgery. At the time of writing, the patient had been followed up for 18 months. Regular follow-up included pelvic ultrasonography every 3 months, pelvic MRI every 6 months, and serum tumor markers (CA125 and CA199) testing every 3 months. No clinical, radiological, or biochemical evidence of recurrence has been detected during the follow-up period.

## Discussion

PJS is an autosomal dominant hereditary disorder primarily characterized by gastrointestinal hamartomatous polyps and mucocutaneous pigmentations, along with a well-established association with increased risks of systemic malignancies (4). Gynecological manifestations, though recognized as important extrapulmonary complications of PJS, are relatively rare when compared with gastrointestinal involvement, and the concurrent occurrence of LEGH and SCTATs in a single PJS patient has rarely been reported in the literature. This case study thus provides valuable insights into the phenotypic heterogeneity of PJS-related gynecological pathology and highlights key considerations for clinical management.

The tumor characteristics of female patients with PJS include SCTATs, ovarian mucinous tumors, and MDA (5). SCTATs account for the majority of ovarian tumors in patients with PJS and is thought to occur in almost all female patients with PJS (6). Malignant transformation is estimated to occur in approximately 20% of cases (7). The pathological hallmark of SCTATs, annular tubules surrounding hyaline eosinophilic cores, and the characteristic immunohistochemical profile (positivity for calretinin, inhibin-A, and SF-1, and negativity for ER and PR) were consistent with these findings, confirming the benign nature of the ovarian lesion in this patient (8). Internal examinations and transvaginal (transabdominal) ultrasound examinations are recommended at 1-year intervals from 18 to 25 years to monitor the ovary and uterus, but CA125 is not recommended (5). However, the effectiveness of periodic surveillance is unclear, and there is concern that screening and surveillance at a young age may increase unnecessary surgical treatment of benign tumors.

MDA is a subtype of hyperdifferentiated gastric-type mucinous adenocarcinoma that is often difficult to differentiate from benign lesions. However, it is a malignant tumor that invades the deep cervix and paracervical connective tissue at an early stage, resulting in lymph node metastasis and poor prognosis (9). SCTAT-induced hyperestrogenism is thought to cause LEGH (10). LEGH is a rare cervical epithelial lesion that is closely associated with PJS, with approximately 30–50% of LEGH cases being reported in patients with PJS or *STK11* mutations (3).

Patients with PJS are also at increased risk of endometrial abnormalities, including atypical hyperplasia and endometrial cancer. The patient’s history of endometrial atypical hyperplasia may represent another component of PJS-related gynecological involvement. Therefore, endometrial evaluation should also be integrated into routine gynecological surveillance for female patients with PJS, including imaging assessment and hysteroscopic biopsy when clinically indicated.

The rarity of concurrent occurrence of LEGH and SCTATs in PJS may be attributed to the low overall incidence of each lesion: LEGH has an estimated prevalence of <0.1% in the general population, and SCTATs represent <1% of all ovarian tumors (10). In patients with PJS, the prevalence of LEGH is approximately 30–50%, and SCTATs are detected in approximately 30–60% of affected females. Although each lesion is relatively common in PJS, concurrent LEGH and SCTATs remain extremely rare, with only a few case reports documented in the literature. This highlights the unique phenotypic spectrum of this case.

The young age of this study patient (25 years) further emphasizes the need for early and comprehensive gynecological screening in PJS patients, even in those without typical gynecological symptoms (e.g., abnormal uterine bleeding). Existing clinical guidelines for PJS recommend annual pelvic ultrasound and cervical cytology starting from early adulthood for screening gynecological malignancies. In a systematic review of Japanese patients with PJS, case reports have been accumulated from patients aged ≥20 years, and surveillance from a young age is important (11). Experts recommend that cervical smears using liquid-based cytology be conducted every 2–3 years starting at age 21 years. Lack of cytological atypia indicates that cytology is not sensitive, and cervical histology, transvaginal (transabdominal) ultrasound, and pelvic MRI should be considered (12, 13). However, this case highlights the limitations of routine screening strategies, namely LEGH is often asymptomatic (as in this patient, who presented only with watery vaginal discharge) and may not be detected by cervical cytology due to its deep glandular location, while SCTATs may present as a small adnexal mass that is easily missed by conventional ultrasound. In this patient, MRI played a crucial role in identifying the multilocular cystic cervical lesion and characterizing the adnexal mass, and PET/CT provided additional information on metabolic activity to exclude malignant progression. MRI has proven essential in distinguishing LEGH from other benign cervical cystic lesions and detecting precursor conditions, such as atypical LEGH, before progression to G-EAC. A hallmark MRI finding for LEGH is the “cosmos pattern,” featuring centrally clustered microcysts surrounded by macrocysts, with 95.5% specificity when combined with T1-weighted imaging (14). Based on this case and existing published evidence, the study suggests that intensified gynecological surveillance may be considered for female patients with PJS, including: (1) pelvic MRI every 2–3 years to detect deep glandular or adnexal lesions, (2) proactive cervical biopsy or hysteroscopy for suspicious imaging findings, even with normal cytology, and (3) long-term follow-up for patients with LEGH. Since this recommendation is based on a single case and limited literature, further large-scale cohort studies are warranted to validate the optimal surveillance strategy. Additionally, genetic testing for *STK11* mutations should be considered in patients with unexplained LEGH or SCTATs, as this may prompt the diagnosis of undiagnosed PJS and initiate appropriate systemic surveillance for gastrointestinal polyps and other malignancies.

PJS patients often require multidisciplinary care involving gastroenterologists, gynecologists, pathologists, and genetic counselors due to the systemic nature of the disease. In this case, the patient had a history of intestinal polyps, duodenal adenocarcinoma, and endometrial atypical hyperplasia, which necessitated coordination between gastroenterological and gynecological teams for surgical planning. Cone biopsy is effective for pathological diagnosis. Simple hysterectomy is indicated as surgical treatment for LEGH; however, meticulous follow-up is also an option, especially for young patients, because the rate of malignant transformation has been reported as 1–2%. For LEGH patients who choose follow-up, worsening cytology results and an increase in lesion size are important signs of malignant change in LEGH for safe follow-up (13). The decision to perform a hysterectomy was based on the patient’s lack of fertility desire and her complex medical history, highlighting the importance of individualized treatment decisions in PJS patients.

Pathological diagnosis also relied on close collaboration between surgical pathologists and clinicians in the differential diagnosis of LEGH, including MDA and G-EAC, and immunohistochemical staining (e.g., MUC-6 positivity and low Ki-67 index) was critical to confirm the benign nature of the lesion (3). Similarly, the diagnosis of SCTAT required distinction from other ovarian sex cord-stromal tumors (e.g., granulosa cell tumor), which was facilitated by their characteristic morphological and immunohistochemical features (15).

This report has several limitations. First, as a single-case study, the findings may not be generalizable to all PJS patients, and larger cohort studies are needed to validate the incidence and clinical outcomes of concurrent LEGH and SCTATs. Second, the follow-up period was relatively short (18 months), and long-term surveillance is required to assess the risk of late recurrence, especially in SCTATs.

## Conclusion

This case describes a rare instance of concurrent LEGH and SCTATs in a young PJS patient, expanding the phenotypic spectrum of gynecological manifestations in PJS. The findings emphasize the need for optimized gynecological surveillance strategies in PJS patients, including the integration of advanced imaging (e.g., MRI) and proactive biopsy to detect early precursor lesions. Multidisciplinary collaboration between clinicians, pathologists, and genetic counselors is essential for accurate diagnosis, individualized treatment, and long-term management. Further research is warranted to clarify the underlying mechanisms of concurrent gynecological lesions in PJS and to refine screening guidelines for this high-risk population.

## Data Availability

The original contributions presented in the study are included in the article/supplementary material, further inquiries can be directed to the corresponding authors. Written informed consent was obtained from the individuals for the publication of any potentially identifiable images or data included in this article.
